# The effect of tuberculosis and antiretroviral treatment on CD4+ cell count response in HIV-positive tuberculosis patients in Mozambique

**DOI:** 10.1186/1471-2458-12-670

**Published:** 2012-08-17

**Authors:** Miranda Brouwer, Paula Samu Gudo, Chalice Mage Simbe, Paula Perdigão, Frank van Leth

**Affiliations:** 1 Health Alliance International, Technical Assistance Unit, Maputo, Mozambique; 2 Ministry of Health, National TB Programme, C.P. 264 Av. Eduardo Mondlane/Salvador Allende, Maputo, Republica de Moçambique; 3 Ministry of Health, Provincial Health Directorate Manica Province, Chimoio, C.P. 264 Av. Eduardo Mondlane/Salvador Allende, Maputo, Republica de Moçambique; 4 Independent chest physician, Maputo, Mozambique; 5 Department of Global Health, Academic Medical Center, University of Amsterdam, Amsterdam Institute for Global Health and Development, Pietersbergweg 17, 1105, BM, Amsterdam, The Netherlands; 6 KNCV Tuberculosis Foundation, Postbus 146, 2501, CC, The Hague, The Netherlands

## Abstract

**Background:**

Tuberculosis (TB) presents a serious problem in Mozambique. HIV prevalence among TB patients is estimated at 47%. A delay in having their first CD4+ cell count could lead to a missed opportunity for ART initiation due to a CD4+ cell increase above the cut-off caused by TB treatment. The objective is to describe CD4+ cell response during TB treatment and quantify the effect of TB treatment and ART on this response.

**Methods:**

All new HIV + adult TB cases in 2007 from three TB clinics in Mozambique were included. Data on TB diagnosis and treatment and HIV parameters were collected. A general mixed model was used for CD4+ cell count response.

**Results:**

338 HIV + patients were notified and 252 (75%) were included in the analysis. Using TB medication was not independently associated with the CD4+ count response (19 cells/mm^3^; 95% CI: -40 to 79; p = 0.529). ART-use was associated with statistically significantly higher CD4+ cells compared to no ART-use (81 cells/mm^3^; 95% confidence interval (CI): 12 to 151; p = 0.022).

**Conclusion:**

In this study, no independent effect of TB treatment on CD4+ cell count was found. HIV-infected TB patients on ART had a significantly higher CD4+ cell count than those not receiving ART. CD4+ cell counts for patients not on ART at TB treatment start, remained below the cut off for initiating ART during the first three months of TB treatment; therefore some delay in getting the first CD4+ cell count would not lead to missing the opportunity to start ART.

## Background

Tuberculosis (TB) presents a serious problem in Mozambique with case notifications rising dramatically since the start of this century. The World Health Organization (WHO) estimated the incidence of all forms of TB in Mozambique in Mozambique at the time of the study (2007) at 431 per 100.000 population [1]. The increase in TB notifications is partly driven by the Human Immunodeficiency Virus (HIV) epidemic [2]. The national HIV prevalence is estimated at 15%, based on antenatal sentinel surveillance among pregnant women 15 to 49 years of age [3]. WHO estimated the HIV prevalence in adult TB cases at 47% in 2007 [1].

In Sub Saharan Africa, people unaware of their HIV-infection present often to the health care services with TB as the first AIDS defining illness. Several studies found that TB clinics are well positioned to identify new HIV-infected individuals and to provide access to HIV services [4,5].

Following international recommendations, Mozambique started implementing TB-HIV collaborative activities in 2006 [6]. TB treatment staff provide HIV counselling and testing, and offer co-trimoxazole preventive therapy (CPT) at the TB clinic to HIV-infected TB patients. They refer co-infected patients to HIV services for further care and treatment, including antiretroviral therapy (ART). According to the 2006 national guidelines, the timing of ART initiation in relation to TB treatment depends on the level of immunosuppression [7]. Patients with a CD4+ cell count less than 200 cells/mm3, should start ART as soon as possible, and in those with a CD4 + cell count between 200 and 350 cells/mm3 ART is delayed until the first two months of TB treatment are completed. At the end of 2009, WHO published new recommendations to start ART as soon as possible in TB-HIV co-infected patients regardless of their immunosuppression [8]. At the same time, the Ministry of Health in Mozambique published new HIV treatment guidelines that had not yet incorporated the new WHO recommendations [9]. These new Mozambican guidelines are still valid presently and the start of ART in co-infected patients still depends on the level of immunosuppression, though the lower level is of the CD4+ cell count is 250 cells/mm3 compared to 200 cells/mm3 in the 2006 guidelines.

Several studies described an increase in CD4+ cell count during TB treatment for non-immune compromised TB patients [10,11]. CD4+ cell response during TB treatment in HIV-infected TB patients is less clear and only a few studies addressed this question. One South African study showed a significant increase of CD4+ cell count after 3 month of TB treatment. Another South African study of HIV-infected TB patients did find an increase in CD4+ cell count during TB treatment, though this was not statistically significant [12]. In both these studies, ART was not available to the participants.

In Mozambique, not all health facilities delivering HIV services have equipment for the assessment of CD4+ cells. Therefore, newly diagnosed HIV-infected TB patients may experience a delay in having their first CD4+ cell count result available. Should the CD4+ cell count during TB treatment increase in the HIV-infected TB patients as in non-immune compromised TB patients, the CD4+ cell count might become higher than the cut-off value for initiating ART. An opportunity for start of ART would be missed.

The objective of this study was to describe the CD4+ cell count response during TB treatment and to quantify the effect of TB treatment and ART on the CD4+ cell count response. Through the CD4+ cell count response we assessed whether a risk exists for missing an opportunity to start ART in the routine setting of Mozambique due to late CD4+ cell count availability in HIV-infected TB patients, and the prioritization of ART for TB-HIV co-infected patients with the lowest CD4+ cell counts.

## Methods

### Ethics statement

The National Bio-ethic Committee of the Ministry of Health of Mozambique and the Institutional Review Board of the University of Washington in Seattle, USA, approved the study protocol. Both ethics committees approved that informed consent was not obtained as the study was based on routinely collected data.

### Study design and setting

We performed a retrospective observational study in three purposely-selected health facilities in Manica province, Mozambique. Selection criteria were the presence of both TB and HIV treatment services in the same facility and at least 150 TB patients notified in 2007. One facility was an urban health centre in the provincial capital; the other two were rural health facilities about 20 and 90 kilometres from the provincial capital. Within these clinics, we collected the information on HIV disease parameters of all new notified TB patients of 16 years and older with a positive HIV test recorded in the TB register from January to December 2007.

In Mozambique, smear microscopy is the main TB diagnostic. In the participating facilities, diagnosis of sputum smear-negative and extrapulmonary TB occurs mainly on clinical assessment and hardly ever on radiology. All new adult TB patients receive a standard course of TB treatment consisting of two months isoniazid, rifampicin, ethambutol and pyrazinamide followed by 4 months isoniazid and rifampicin. The standard first line ART regimen consists of two nucleoside reverse-transcriptase inhibitors, lamivudine and stavudine, with either the non-nucleoside reverse transcriptase inhibitor (NNRTI) nevirapine or efavirenz. The national guidelines recommend switching from neviripine to efavirenz in patients that receive a rifampicin containing treatment regimen [7].

### Data collection

The facility’s TB supervisor collected the data of the 2007 cohort using standard data collection forms in July and August 2009. Data collected from the TB register included: age, sex, type and category of TB, treatment regimen, start date of TB treatment, initial smear examination result, HIV test result and TB treatment outcome. If the treatment outcome was death, its date was recorded.

We identified the HIV record of the HIV-positive TB cases through the unique HIV patient number if recorded in the TB register. In addition, local staff familiar with the patients identified some HIV patient records. If these methods did not lead to identification of the patient record, we searched the electronic HIV-database using the patient’s name and age taken from the TB register. If the data matched, we took the unique HIV patient number from the electronic database and used it to locate the HIV patient record. We limited the identification of the HIV patient record to those HIV-positive TB patients registered with the HIV services in the same health facility.

We collected available CD4+ cell count results in the 6 months TB treatment period, the date of these results, the start date for ART and the ART regimen from the HIV patient record.

### Statistical analysis

We entered the data in EpiData version 3.1 and performed descriptive analysis with EpiData Analysis V2.2.1.171. We used STATA version 11 (StataCorp, College Station, Texas, USA) for analysis of the CD4+ cell counts.

We modelled the evolution of the CD4+ cell count during TB treatment using a mixed effect model. This model deals adequately with repeated measurements of the outcome variable [13]. The model incorporates estimated values for missing data based on all other available data. With this model we used optimally all available CD4+ cell counts including all patients with at least one CD4+ cell count in the model, regardless of the number of missing values these patients have. We used a random intercept model with an independent covariance structure for estimation of the CD4+ cell count over time. We compared the mean CD4+ cell count for the time updated variables of TB treatment use and ART use. In addition we included age and sex in the model as potential confounding variables. The model used the absolute CD4+ cell count values to estimate the effect of TB treatment and ART on CD4+ cell response. We assumed that once a patient starts ART, the patient continues ART until the end of the observation period. We did not adjust for the type of NNRTI because there is no evidence that there is a differential CD4+ count increase when comparing a nevirapine-based or efavirenz-based regimen [14].

### Definitions

We defined the baseline CD4+ cell count at the start of TB treatment as the CD4+ count closest to the start of TB treatment within a window of 12 weeks before until 2 weeks after the start of TB treatment. We allocated all other CD4+ cell counts to a single fixed time-point with a window ranging from 2 to 6 weeks. If multiple CD4+ cell counts were available for a specific time-point, we included the one closest to the midpoint of the time window in the analysis.

To determine whether the CD4+ cell count was obtained while using TB treatment, we used WHO standard treatment outcomes to define the end of TB treatment [15]. The end of TB treatment for treatment ‘success’ (cured or treatment completed) was 180 days after start of TB treatment. For ‘failure’ (smear positive after five months for sputum smear positive cases), the end of treatment was 150 days (5 months) after start. For ‘default’ (interrupted treatment for two or more consecutive months), ‘transfer out’ (transferred to another TB unit with unknown treatment outcome), or ‘unknown’ the end of TB treatment was 90 days after the start date. For patients who died, TB treatment ended at the date of death or 90 days after start of TB treatment if unknown.

## Results

From January 1st until December 31st 2007, 591 new TB patients were notified. Of these, 478 (81%) were tested for HIV and 338 (71%) had a positive test. Of these, 274 (81%) were registered with the HIV services in the same facility. HIV patient records were identified for 256 (93%) patients. Four patients were excluded from the analysis due to an identification mismatch, leaving 252 patients for analysis. All patients started TB treatment. Patient characteristics and the ART status are given in Table 1.

**Table 1:** Demographical and clinical characteristics for HIV-positive new TB patients

	**Registered at HIV services and record available**	**Not registered at HIV services or record not available**	**Total**
Total	252	82	334
Sex *			
Male	107 (43%)	45 (55%)	152 (46%)
Female	144 (57%)	37 (45%)	181 (54%)
Median (IQR^†^) age in years	32 (26–39)	33 (24.8-40.3)	32 (26–39.3)
Type of TB			
Smear-positive Pulmonary TB	156 (62%)	49 (60%)	205 (61%)
Smear-negative Pulmonary TB	79 (31%)	28 (34%)	107 (32%)
Extra Pulmonary TB	17 (7%)	5 (6%)	22 (7%)
TB Treatment outcome			
Cure and treatment completed	196 (78%)	63 (77%)	259 (78%)
Died	52 (21%)	17 (21%)	69 (21%)
Other	4 (2%)	2 (2%)	6 (2%)
ART use			
no ART during the study period	67 (27%)	N/A^&^	
started ART before TB diagnosis	81 (32%)	N/A	
started ART during TB treatment	86 (34%)	N/A	
started ART after TB treatment	18 (7%)	N/A	

* One patient did not have the sex recorded in the TB register.

† IQR = Inter Quartile Range.

& N/A = Not applicable.

**Figure 1:**
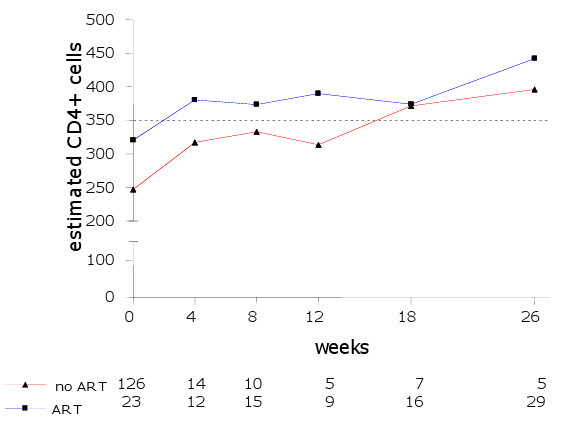
**Estimated CD4+ cell counts by ART use.** Legend: The numbers below the figure show the CD4+ cell counts available per time-point.

Of the 252 patients available for analysis, 25 (10%) had not a single CD4+ count recorded and 16 (6%) had only CD4+ counts before the period defined as the start of TB treatment. For the remaining 211 (84%) patients, a total of 271 CD4+ counts were available at the start of or during TB treatment. Figure 1 shows the number of available CD4+ cell counts at each time-point.

A baseline CD4+ cell count was available for 149 patients with 48 (32%) and 23 (15%) taken while the patient received TB treatment or ART respectively. Of the 122 CD4+ count results after the start of TB treatment, 81(66%) were obtained while the patient was on ART.

Over the full observation period of 6 months, using TB treatment was not statistically significant associated with the CD4+ cell count response. Patients using TB treatment had a CD4+ cell increase of 19 cells/mm3 (95% CI: -50 to 79; p = 0.529) compared to patients not receiving TB treatment. ART use was statistically significantly associated with CD4+ cell response during the observation period. Patients using ART had a CD4+ cell increase of 81 cells (95% CI: 12 to 151; p = 0.222) compared to patients not using ART.

The CD4+ cell count response during TB treatment by ART status is shown in Figure 1.

## Discussion

In this study, TB treatment in TB-HIV co-infected patients had no significant effect on CD4+ cell count. The evolution of CD4+ cell count is mainly driven by ART use. The average CD4+ cell count for patients not on ART remained below the cut-off for initiating ART of 350 cells/mm^3^ during the first 12 weeks of TB treatment. Therefore, a delayed assessment of the first CD4+ cell count in itself would probably not lead to missing an opportunity to start ART based on the cut off of 350 cells/mm3. This finding is relevant in the Mozambican setting where not all ART treatment facilities are capable of performing CD4+ cell counts and prescription of ART is prioritized. Health facilities sent blood samples for CD4+ cell count to another laboratory daily or weekly, depending on the distance between the sending and receiving facilities.

This study showed a small increase in CD4+ cell count during TB treatment in both patients on ART and patients not on ART as has been described in non-immune compromised TB patients [10,11]. In other studies the CD4+ cell count in HIV-infected TB patients not on ART did not increases [12,16]. It seems that the immune response in HIV-infected TB patients not on ART is variable.

This was a retrospective study based on routine data and as such has several limitations. First, bias may have occurred by not including patients who received HIV treatment at another health facility. These patients may have been treated differently or adherence may have been different. This would have influenced treatment outcome and CD4+ cell count. All health care facilities in Mozambique follow the same national guidelines and as such the chance of a difference in treatment strategy is unlikely. Furthermore, the characteristics for patients whose clinical record was identified were very similar with those from patients without an HIV record (Table 1).

Second, not all TB patients in this study had an HIV-test or the result recorded in the TB register. Therefore, not all HIV-infected TB patients were included. Given the high testing rate of more than 80%, it is unlikely that the non-availability of the HIV-test result would markedly bias the results of the study.

Third, this study took place in three health facilities in a single province of Mozambique. The results may be different in other areas in Mozambique. However, we believe that the situation in Manica does not differ much from that in other provinces in the country at the time of the study, apart from the larger cities where more ART facilities are available. There is also more specialist care available in the larger cities.

Fourth, despite a considerable amount of patients, the number of available CD4+ cell counts per patient was small, reflecting the indications in the national guidelines as to when to perform this test. The use of the mixed model allowed us to use all available data and was therefore the recommended methodology for our data set.

Fifth, about one third of the patients that used ART during TB treatment started their ART before the start of TB treatment and potentially had incident TB while using ART. Emerging evidence shows that CD4+ cell count response is smaller in these patients [17]. However, earlier evidence showed a similar CD4+ cell count response in both patients with prevalent and those with incident TB compared to patients on ART without TB [18]. We cannot completely rule out a potential underestimation of the effect of TB treatment in our study. However, the majority of patients did not have this incident TB and we are confident that our results are valid.

Despite these limitations, we consider the results relevant and important because limited data are available on CD4+ cell count response in cohorts of TB patients.

The presently used HIV treatment guidelines in Mozambique are not yet in line with the WHO recommendation to initiate ART in HIV-infected TB patients as soon as possible after the start of TB treatment irrespective of the CD4+ cell count [8]. The present study supports this recommendation as patients on ART had a much better immune restoration than those not on ART. However, like in Mozambique, these WHO guidelines have not yet been implemented everywhere.

Also, many countries with a high burden of HIV struggle to maintain all HIV-infected patients on ART. Lack of funding may lead to stock outs of antiretroviral drugs at facility level [19]. Therefore countries may wish to prioritize new initiations of ART to those most in need. For HIV-infected TB patients, the CD4+ cell count provides a tool to prioritize. This study shows that obtaining a sample for CD4+ cell count assessment in the first 12 weeks of TB treatment will be a reliable indicator for the need to initiate ART, since this measurement is not influenced by concurrent TB treatment. The opportunity for identifying the HIV-infected TB patients most in need of ART is unlikely to be missed.

## Conclusion

In this study the higher CD4+ cell count level during TB treatment in HIV-infected TB patients is due to ART use, and not influenced by TB-treatment. Therefore, these findings are a strong argument to implement the recent WHO recommendation to start ART as soon as possible in HIV-infected TB patients irrespective of their CD4+ cell count. Should countries wish or need to prioritize new ART initiations to those most in need, a CD4+ cell count result obtained in the first 12 weeks of TB treatment provides a good reflection of the immune status at the start of TB treatment. This holds for Mozambique and probably for other similar settings as well.

A prospective study will provide better insight to the question of the CD4+ cell count response during TB treatment and the effect of TB treatment and ART on this response.

## Competing interests

The authors declare that they have no competing interests.

## Authors’ contributions

MB conceived the study, participated in the design of the study, had the overall coordination of the data collection, participated in the statistical analysis and drafted the manuscript. PSG conceived the study, participated in the design of the study and helped to draft the manuscript. CMS participated in the design of the study and coordinated the data collection. PP conceived the study, participated in the design of the study and helped to draft the manuscript. FL participated in the design of the study, participated in the statistical analysis and helped to draft the manuscript. All authors read and approved the final manuscript.

## Pre-publication history

The pre-publication history for this paper can be accessed here:

http://www.biomedcentral.com/1471-2458/12/670/prepub
